# Effects of road salt on microbial communities: Halophiles as biomarkers of road salt pollution

**DOI:** 10.1371/journal.pone.0221355

**Published:** 2019-09-04

**Authors:** Wolf T. Pecher, M. Emad Al Madadha, Priya DasSarma, Folasade Ekulona, Eric J. Schott, Kelli Crowe, Bojana Stojkovic Gut, Shiladitya DasSarma

**Affiliations:** 1 Institute of Marine and Environmental Technology, Department of Microbiology and Immunology, University of Maryland School of Medicine, Baltimore, Maryland, United States of America; 2 Yale Gordon College of Arts and Sciences, University of Baltimore, Baltimore, Maryland, United States of America; 3 Department of Pathology, Microbiology and Forensic Medicine, School of Medicine, The University of Jordan, Amman, Jordan; 4 Institute of Marine and Environmental Technology, University of Maryland Center for Environmental Science, Baltimore, Maryland, United States of America; University of Minnesota Twin Cities, UNITED STATES

## Abstract

Increased use of salting to de-ice roadways, especially in urban areas, is leading to elevated salinity levels in soil as well as surface- and ground water. This salt pollution may cause long-term ecological changes to soil and aquatic microbial communities. In this study, we examined the impact on microbial communities in soils exposed to urban road salt runoff using both culturing and 16S amplicon sequencing. Both methods showed an increase in halophilic Bacteria and Archaea in samples from road salt-exposed areas and suggested that halophiles are becoming persistent members of microbial communities in urban, road salt-impacted soils. Since salt is a pollutant that can accumulate in soils over time, it is critical to begin assessing its impact on the environment immediately. Toward this goal, we have developed a facile semi-quantitative assay utilizing halophilic microbes as biomarkers to evaluate on-going salt pollution of soils.

## Introduction

**Fig 1:**
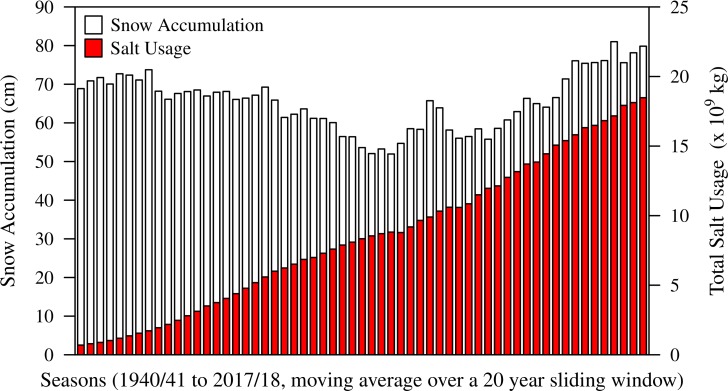
Trends of snow accumulation and salt usage for de-icing roadways. Average snow accumulation in Central Park, New York, and salt usage for de-icing for the USA are shown using a 20-year sliding window starting in the winter season of 1940/41. The sliding window was chosen to minimize annual fluctuations which would otherwise mask the long-term trend. Snow accumulation data were obtained from the National Weather Service [13], and salt usage data from the U. S. Geological Survey [14].

Salt pollution is a long-term environmental concern, potentially threatening soil, lake, and stream ecosystems, and groundwater supplies, as well as coastal regions [1–7]. In particular, road salt crystals and brines used to de-ice roads have become major sources of salt pollution in cold regions of the world, while their use has increased substantially over the past decades [2, 8, 9] (Fig 1). Even as the frequency of snow storms and total accumulation have diminished due to climate change, larger and more intense winter precipitation events are leading to increased demand for application of road salt. Global climatic trends are predicted to further increase these extreme events, including record-breaking storms [10–12]. This trend will likely continue, especially in north-central USA, where climate change models predict an increase in the intensity of snowfall and ice storm events [12].

Salt pollution causes damage to roadside vegetation, material goods, and infrastructure, including roads and bridges, as well as water systems. Salt removal from drinking water supplies requires installation of expensive desalination systems [15]. In the 2017/18 winter season, nearly 22 million metric tons of road salt was applied for de-icing in the USA [16]. Estimated damage due to road salt application was between $803 and $3,401 per ton applied [15] resulting in annual costs of up to $75 billion in the USA.

Negative impacts of salt pollution on human health primarily include the effects from rising sodium concentrations in fresh-water drinking reservoirs, where elevated sodium levels contribute to hypertension and consequently an increased burden on health care systems [17–19].

Salt pollution also affects biodiversity and the composition of microbial communities [20–22] which can indirectly impact human health. Increasing salt levels in freshwater systems favor salt-tolerant cyanobacteria, including some that can produce harmful toxins [22–26]. Furthermore, rising salt levels have been linked to the increased release of toxins by cyanobacteria into the air and water [24, 25, 27, 28]. Consumption of or exposure to these toxins is known to cause liver damage and possible carcinogenic effects [27, 29–33]. Moreover, harmful blooms can adversely affect the tourism and food industries, causing additional economic hardship [34].

Other negative effects of salt pollution include changes in soil biogeochemistry, composition, and characteristics such as decreased aeration and decreased soil permeability with increased erosion [22]. In aquatic environments, salt pollution may impact stratification of lakes and shift biological communities towards more salt tolerant species and reduction of biodiversity [20–22, 35, 36].

Increased salt concentrations heighten osmotic stress which in turn results in inhibition of non-halophilic microbial species and stimulation of halophilic ones. The most sensitive microbes are non-halophilic species which tolerate less than 0.2 M sodium chloride (NaCl). These microbes become osmotically stressed in the presence of even small increases in salt concentrations and may only survive in pockets of lower salinity or in dormant spore forms. Growth of slight halophiles (tolerating up to 0.85 M NaCl), moderate halophiles (tolerating up to 3.4 M NaCl), and extreme halophiles, which require high salt concentrations for growth and tolerate up to 5.2 M (saturated) NaCl, may be promoted when salt concentrations are substantially [37–39].

Sodium chloride is the primary salt used to de-ice roads in the USA, employing halite crystals from salt mines and solar salterns. These have long been known to be rich sources of halophilic microorganisms, harboring a wide variety of species which includes Bacteria and their spores, Archaea, and some Eukaryotes, such as green algae [37, 40–43]. In this initial study, we address whether any halophilic microorganisms are becoming either seasonal or persistent members of proximal microbial communities and if their presence may be used as indicator and biomarkers for salt pollution.

## Materials and methods

### Sampling and sample sites

**Fig 2:**
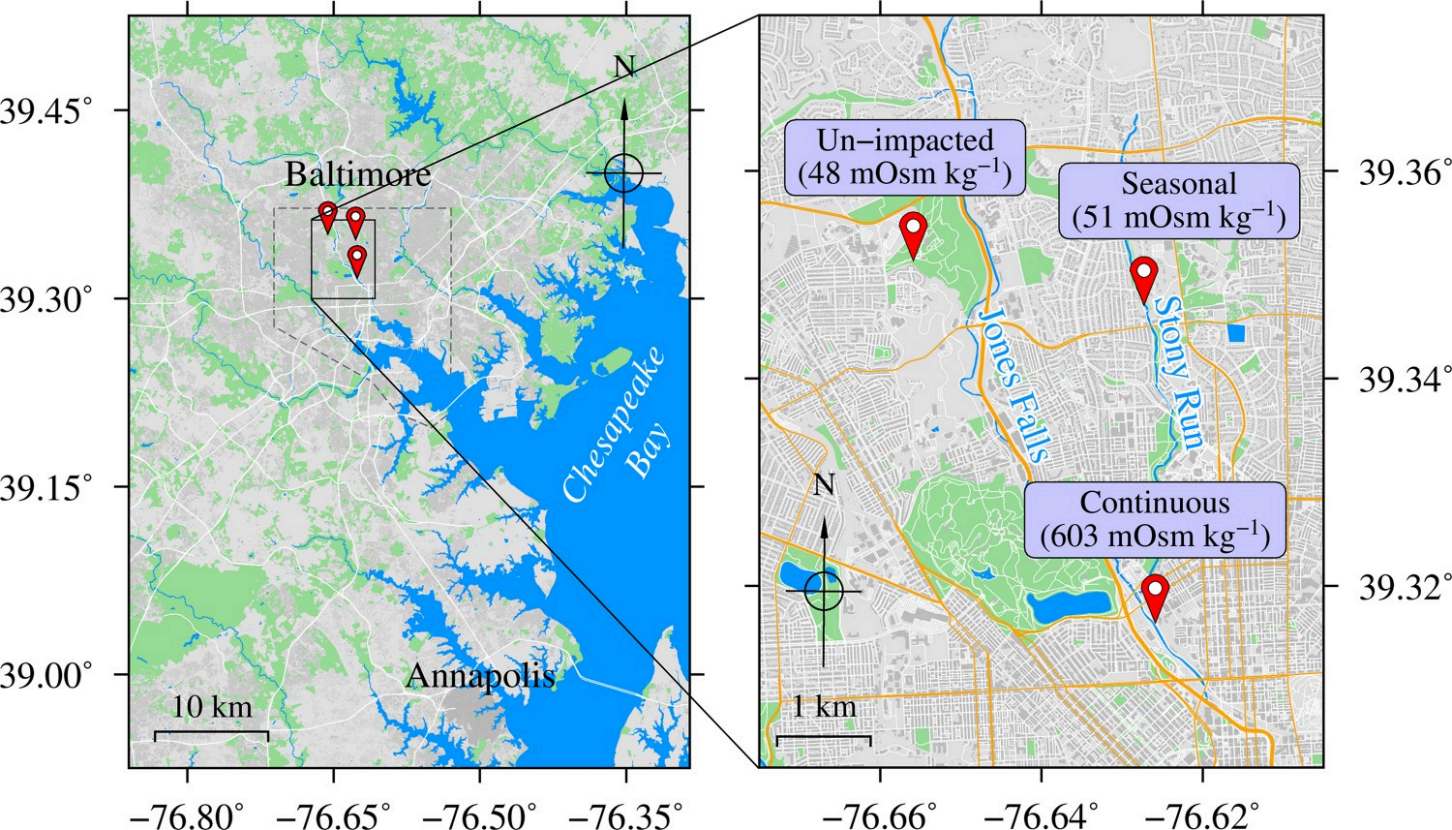
Sampling locations and relative salinity measurements. Sample sites are located in the Jones Falls watershed (MD 8-digit watershed code: 02130904), a sub-watershed of the Patapsco River watershed (MD 6-digit watershed code: 021309). The control un-impacted site (with an osmolality of 48 mOsm kg^-1^) is located in an arboretum on a ridge-top away from any road. The seasonally impacted site (with an osmolality of 51 mOsm kg^-1^) is located in a park receiving runoff from streets in a residential neighborhood, and the continuously impacted site (with an osmolality of 603 mOsm kg^-1^) is located adjacent to a year-round salt storage and distribution facility. The location of the Jones Falls, the main contributor to the Inner Harbor of Baltimore, Maryland, USA, and one of its tributaries, the Stony Run are also indicated. Maps were created using Generic Mapping Tools v. 6.0.0 [44] and GIMP v. 2.8.22. Map data were obtained from Geofabrik GMBH (download.geofabrik.de). Map data OpenStreetMap contributors are licensed under the Open Data Commons Open Database License (ODbL; https://opendatacommons.org/licenses/odbl/1.0/index.html) by the OpenStreetMap Foundation, and the Creative Commons Attribution-ShareAlike 2.0 License (CC BY-SA; https://creativecommons.org/licenses/by-sa/2.0/legalcode).

Soil samples were collected from 3 sites in Baltimore, Maryland, USA. The sample sites are located in the Jones Falls watershed (Maryland (MD) 8-digit watershed code: 02130904) which is a sub-watershed of the Patapsco River watershed (MD 6-digit watershed code: 021309). Based on frequency of road salt exposure, the sites were designated as (1) un-impacted, (2) seasonally, and (3) continuously impacted. The control un-impacted site (longitude: -76.655922°, latitude: 39.351340°, elevation: 126 m) is located within a public arboretum, approximately 30 m south of a service road and atop a gentle ridge top. The site is approximately 1 m higher than the service road. The seasonally impacted site (longitude: -76.627504°, latitude: 39.347162°, elevation: 84 m) is located in a public park that receives runoff from streets of a residential neighborhood that are salted in winter. The continuously impacted site (longitude: -76.625879°, latitude: 39.316416°, elevation: 20 m) is next to a street running parallel to the Jones Falls. An open salt storage and distribution facility of the City of Baltimore was located across the street from the continuously impacted site at the time of sampling. Road salt samples were obtained from the salt storage facility as well as from a salt box in the residential neighborhood close to the seasonally impacted site (Fig 2).

At each location, samples were taken using a sterile spatula from the top 5 cm of soil (or salt pile) from an area about 10 cm in diameter. A total of approximately 40 grams of soil or salt was collected in sterile plastic sample bags or 50 ml conical tubes and stored at 4°C until further processing. For the isolation and culturing of halophiles, soil samples from the seasonally and continuously impacted sites, and salt samples were collected in February 2013. For the quantification and assessment of soil microbial communities, using spot testing, colony counting, and amplicon sequencing of the 16S rRNA gene, soil surface samples were collected from the control un-impacted, the seasonally impacted, and continuously impacted sites in November 2014, before salt was applied (about 8 months after the last salt application), in February 2015 at the end of the salting season, and in June 2015, close to 3 months after the last salt application.

Salt content of soils was estimated by measuring osmolality of water in which soil was suspended. Soil samples collected in winter 2014, were dried at 50°C for 16 hours, followed by suspension in an equal weight of distilled water for 1 hour to dissolve salts. A subsample was centrifuged at 10,000 x *g* for 2 minutes and the osmolality of the supernatant measured in a Wescor VAPRO Vapor Pressure Osmometer model 5520 (ELITech Group, Puteaux, France).

### Enrichment cultures and isolation of halophiles

Five hundred mg of soil or salt samples were resuspended in halophile broth (HB; 80 mM MgSO_4_, 10 mM trisodium citrate, 27 mM KCl, 10 g/L Oxoid peptone, 2.3 μM ZnSO_4_, 0.2 μM MnSO_4_, 0.2 μM (NH_4_)_2_Fe(SO_4_)_2_, 56 nM CuSO_4_, pH 7.2) supplemented with 2 M NaCl or 4.3 M NaCl and incubated at 42°C under constant agitation at 220 RPM in an Innova 44R shaker (New Brunswick, Connecticut, USA) for up to two weeks. Enriched cultures were subsequently streaked onto 2 M or 4.3 M NaCl HB agar plates and incubated at 42°C for up to one week. Single colonies were picked and partial 16S rDNA PCR amplified as described below.

### Quantification of halophiles

We developed a semi-quantitative spot test to estimate the abundance of halophiles in soil samples. To assess the accuracy of the spot test, we also quantified the presence of halophiles by serial dilution plating and counting of colony forming units (CFU). The spot test was intended to be a convenient and relatively rapid semi-quantitative tool. We therefore chose to reduce the incubation time of plates to 48 hours at 37°C, a temperature routinely used by laboratories that culture microorganisms. At sites with larger numbers of halophiles, the faster growing halophiles may not only out-compete the slow growing halophiles, but also potentially overgrow the plates making an accurate colony count difficult.

Five tenths cm^3^ of soil was resuspended in 1 ml HB medium supplemented with 2 M NaCl, and 10-fold serial dilutions (from 1 x 10^0^ to 1 x 10^−3^) prepared. For the spot test, 20 μl of the serial dilutions were spotted in triplicate onto 2 M NaCl HB agar plates in a grid. To quantify halophiles,100 μl of the serial dilutions were plated in triplicate onto 2 M NaCl HB agar plates. Plates were incubated up to 48 hours at 37°C, examined for microbial growth, and CFU counted.

### Nucleic acid extraction

To obtain genomic DNA from microbial isolates, single colonies were picked from HB plates, and grown in 5 ml HB medium supplemented with 2 M or 4.3 M NaCl at 42°C with continuous shaking at 200 RPM in an Innova 44R shaker (New Brunswick, Connecticut, USA). Cells were harvested and DNA extracted as described previously [45]. Genomic DNA from soil and salt samples was extracted using the MO BIO PowerLyzer PowerSoil DNA isolation kit per manufacturer’s recommendation (MO BIO Laboratories, Inc., Carlsbad, California, USA).

### PCR amplification and sequencing

For the identification of isolates, partial 16S rDNA covering the variable regions V4 to V9 was amplified with the primers 515F (5’-GTGCCAGCMGCCGCGGTAA-3’) [46] and 1492R (5’-GGTTACCTTGTTACGACTT-3’) [47]. The PCR reactions (25μl) were carried out with 4% (v/v) dimethyl sulfoxide (Sigma-Aldrich, St. Louis, Missouri, USA), 10 mM Tris-HCl, 50 mM KCl, and 1.5 mM MgCl_2_, 200 μM of each dNTP, 6.25 pM of each primer, and 1.25 U of Taq DNA polymerase (all chemicals from New England Biolabs, Ipswich, Massachusetts, USA). Thermocycler conditions used were 94°C for 2 min, 35 cycles of 92°C for 1 min, 50°C for 45 sec with an extension of 1 sec per cycle, 72°C for 1.5 min, with a final extension at 72°C for 7 min.

PCR products were size separated by electrophoresis on 1% agarose gels and visualized by staining with ethidium bromide (0.5 μg/ml) using a Fotodyne FOTO/Analyst FX system (Fotodyne Inc., Hartland, Wisconsin, USA). Purified PCR products were sequenced using the primers described above. PCR product purification and sequencing services were provided by the Bioanalytical Services Laboratory at the Institute for Marine and Environmental Technology, Baltimore, Maryland, USA.

Sequences were checked for quality and trimmed using trev from the Staden package v. 2.0.0b10 (http://staden.sourceforge.net/) on a Linux Fedora 20 based computer (Red Hat Inc., Raleigh, North Carolina, USA). Trimmed sequences were queried with the Ribosomal Database Project (RDP) Classifier [48] trained against the RDP database 11.3 (released on Sep. 17, 2014; http://rdp.cme.msu.edu/index.jsp) [49].

### Amplicon sequencing of the 16S rRNA gene

Amplicon sequencing of the 16S rRNA gene was performed by the Bioanalytical Services Laboratory at the Institute for Marine and Environmental Technology, Baltimore, Maryland, USA on an Illumina MiSeq platform as per manufacturer’s instructions (Illumina, Inc., San Diego, California, USA). The Illumina protocol uses the primer pair Bakt_341F and Bakt_805R29 [50]. Sequences were processed using the open source software suite mothur (v. 1.39.5) following procedures developed for sequences generated by the Illumina MiSeq platform [51–53]. Briefly, paired-end sequences were assembled with a quality threshold of 20 and contigs aligned against a customized reference database that was created based on the SILVA non-redundant SSU Ref 99 database (SILVA SSU Ref NR 99 v132, released on Dec. 13, 2017, [54]). After removal of poor alignments and chimeras, sequences that were 97% or more identical were binned into operational taxonomic units (OTUs). OTUs were queried against the customized reference database using a pseudobootstrap value of 80. Finally, sequences that could not be classified (i.e., “unknown” sequences), as well as sequences identified as eukaryotes, mitochondria, and chloroplasts were removed prior to further analysis. Subsequent analysis of the sequence dataset was performed in R (v. 3.4.1) [55].

To create a customized reference database that was compatible with mothur, the SILVA SSU Ref NR 99 database was processed using scripts published online [56, 57] with slight modification. Briefly, to retain most archaeal and bacterial SSU sequences, instead of trimming the SILVA SSU Ref NR 99 database to a region covering almost the entire SSU, sequences were trimmed starting at position 201 and ending at position 1000 of the SSU (based on the 16S sequence of *Escherichia coli* [GenBank Acc.# J01859.1]). The resulting reference database retained all archaeal genera and 99.8% of the bacterial genera that are present in the unprocessed SILVA SSU reference database. To ensure high quality alignments of the contigs, the customized reference database was further trimmed to cover only V3 to V5 regions of the SSU. All sequence analyses were performed on an Arch Linux based computer (4–16.7-1-ARCH, https://www.archlinux.org).

### Sequence data availability

Raw sequence data were deposited in the NCBI BioProject database (https://www.ncbi.nlm.nih.gov/bioproject/) with the accession number PRJNA522438 and 16S partial sequences of isolates deposited in GenBank (https://www.ncbi.nlm.nih.gov/genbank/) with accession numbers MK543962 to MK543988, MK543991 to MK543994, and MK543996 to MK544007.

### Statistical analysis

Statistical analysis was performed in R v. 3.4.1[55]. To compare colony counts, a Conover-Iman test was performed using the R package conover.test v. 1.1.5 [58–60]. The Conover-Iman test was chosen over other possible *post hoc* non-parametric tests (such as the Dunn’s test) due to its power [59, 60]. The abundance of selected microbial groups was compared using a Fisher’s exact test (part of the R base package [55]). The Fisher’s exact test was chosen over a χ^2^ analysis as the Fisher’s exact test does not rely on approximations. When *post hoc* pairwise comparisons were performed, P-values were adjusted with the Holm-Bonferroni correction [61]. For all statistical analysis, the significance level α was set to 0.05. Graphs were generated within R using the packages lattice v. 0.20.35 [62], latticeExtra v. 0.6.28[63], grid v. 3.4.1[55], gridExtra v. 2.2.1[64], and with the GNU Image Manipulation Program (GIMP) v. 2.8.22 (http://www.gimp.org) and Inkscape v. 0.92 (https://inkscape.org/en).

## Results

### Isolation and classification of halophilic prokaryotes

Halophiles were isolated by preparing enrichment cultures in HB medium containing either 2 M or 4.3 M NaCl followed by multiple rounds of sub-culturing of single colonies on the same agar plates. Based on differences in colony and cellular morphology, a total of 43 isolates were chosen for classification through 16S rDNA sequence analysis. Twenty isolates came from road salt samples (6 and 14 from the salt storage facility, and the salt box, respectively), and 23 from the soil samples (13 from the continuously and 10 from the seasonally impacted site). DNA prepared from candidate halophilic strains and partial 16S rDNA (covering the variable regions V4 to V9) was amplified by PCR with primers specific to prokaryotes [46, 47] followed by Sanger sequencing. Thirty of the isolates were assigned to 4 bacterial genera in the family *Bacillaceae*: *Thalassobacillus* (14 isolates), *Halobacillus* (8 isolates), *Piscibacillus* (6 isolates), and *Pontibacillus* (1 isolate), and 1 isolate was identified as *Staphylococcus* (family: *Staphylococcaceae*). The remaining 13 isolates belonged to the archaeal genera in the family *Haloarchaeaceae*/*Halobacteriaceae*: *Natrinema* (10 isolates) and *Haloterrigena* (3 isolates). The *Thalassobacillus*, *Halobacillus*, and *Natrinema* species were isolated from both road salt and soil samples (Table 1).

**Table 1:** Comparison between the culture based and metagenomic approaches in detecting halophilic families.

	Culture method				16S amplicon sequencing			
Family	Salt storage facility	Soil, continuously impacted	Salt box	Soil, seasonally impacted	Salt storage facility	Soil, continuously impacted	Salt box	Soil, seasonally impacted
*Haloarchaeaceae/Halobacteriaceae*	**✔**	**✔**	**✔**	**✔**		**✔**	**✔**	**✔**
*Bacillaceae*	**✔**	**✔**	**✔**	**✔**		**✔**	**✔**	**✔**
*Staphylococcaceae*			**✔**				**✔**	**✔**

Culturing was first utilized to detect members of halophile families shown in the table as markers for salt pollution. 16S amplicon sequencing data were later obtained and were confirmatory of culturing results, with the exception of detection of *Haloarchaeaceae*/*Halobacteriaceae* and *Bacillaceae* families in the salt sample of the salt storage facility. Checkmarks indicate which families were detected at the site.

### Spot test for a semi-quantitative assessment of halophiles

**Fig 3:**
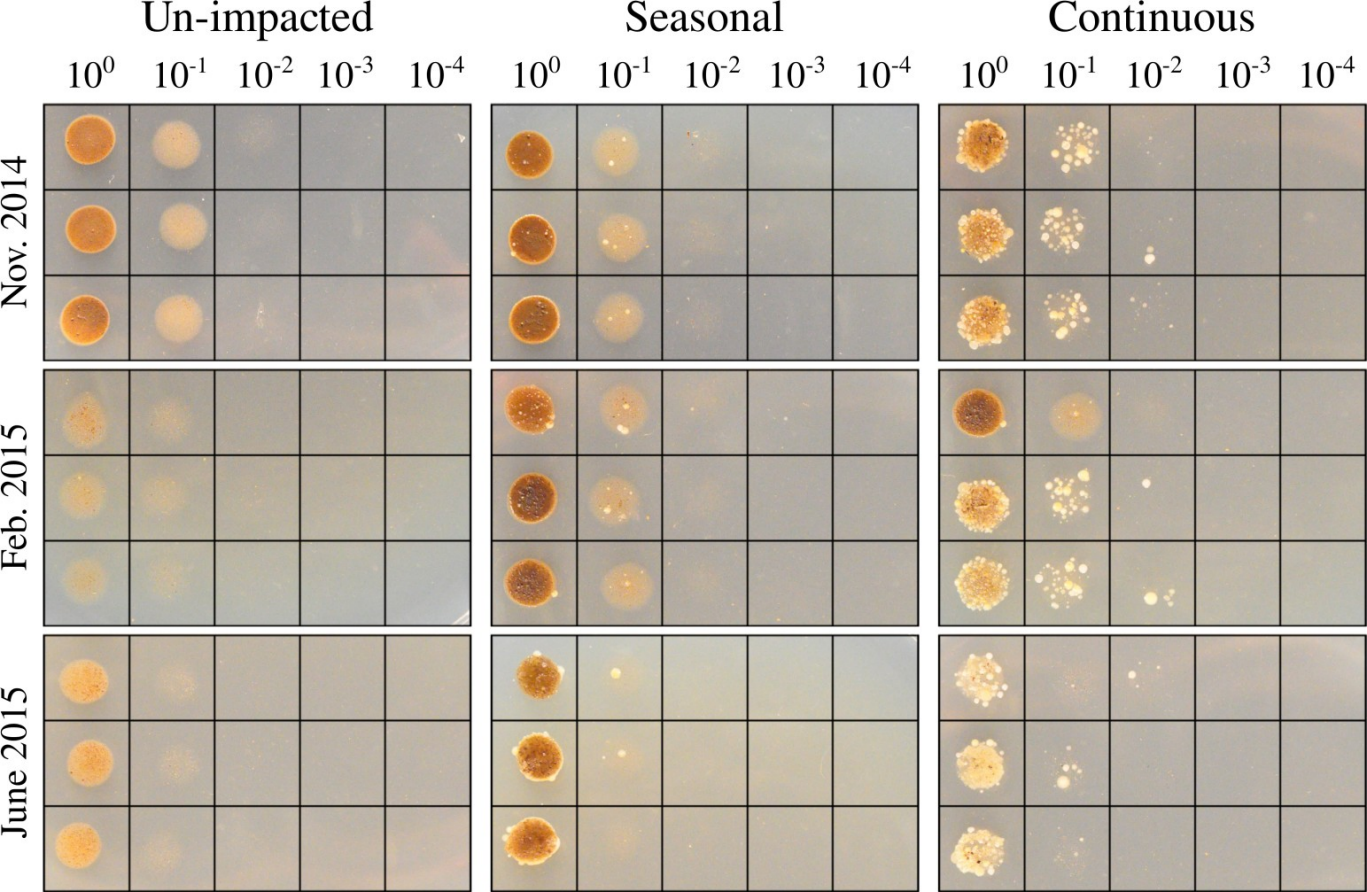
Spot test for a semi-quantitative assessment of halophiles. Twenty μl of serially diluted soil suspensions were spotted in triplicate onto HB agar plates supplemented with 2 M NaCl and incubated for 48 hours at 37°C. Note that the brown to light brown spots seen at the lower dilutions (10^0^ to 10^−1^) are residual insoluble materials from the soil suspensions. Colonies appear as cream-colored dots. Un-impacted soil shows no growth on the high salt agar, while seasonally and continuously impacted sites show halophile growth. Continuously impacted sites show the highest growth levels. Summer season shows the least amount of growth across all samples.

We developed and evaluated a semi-quantitative spot test to assess the presence of halophiles in soil samples. Soil was suspended in 2 M NaCl HB broth, spotted in triplicate on 2 M NaCl HB media plates, and incubated for 48 hours at 37°C. No growth was observed from samples collected from the un-impacted site. The salt-impacted sites showed growth even at the higher dilutions. The continuously impacted site was found to harbor at least 5 to 10 times more halophiles than the seasonally impacted sites (Fig 3).

### Quantification of halophiles

**Fig 4:**
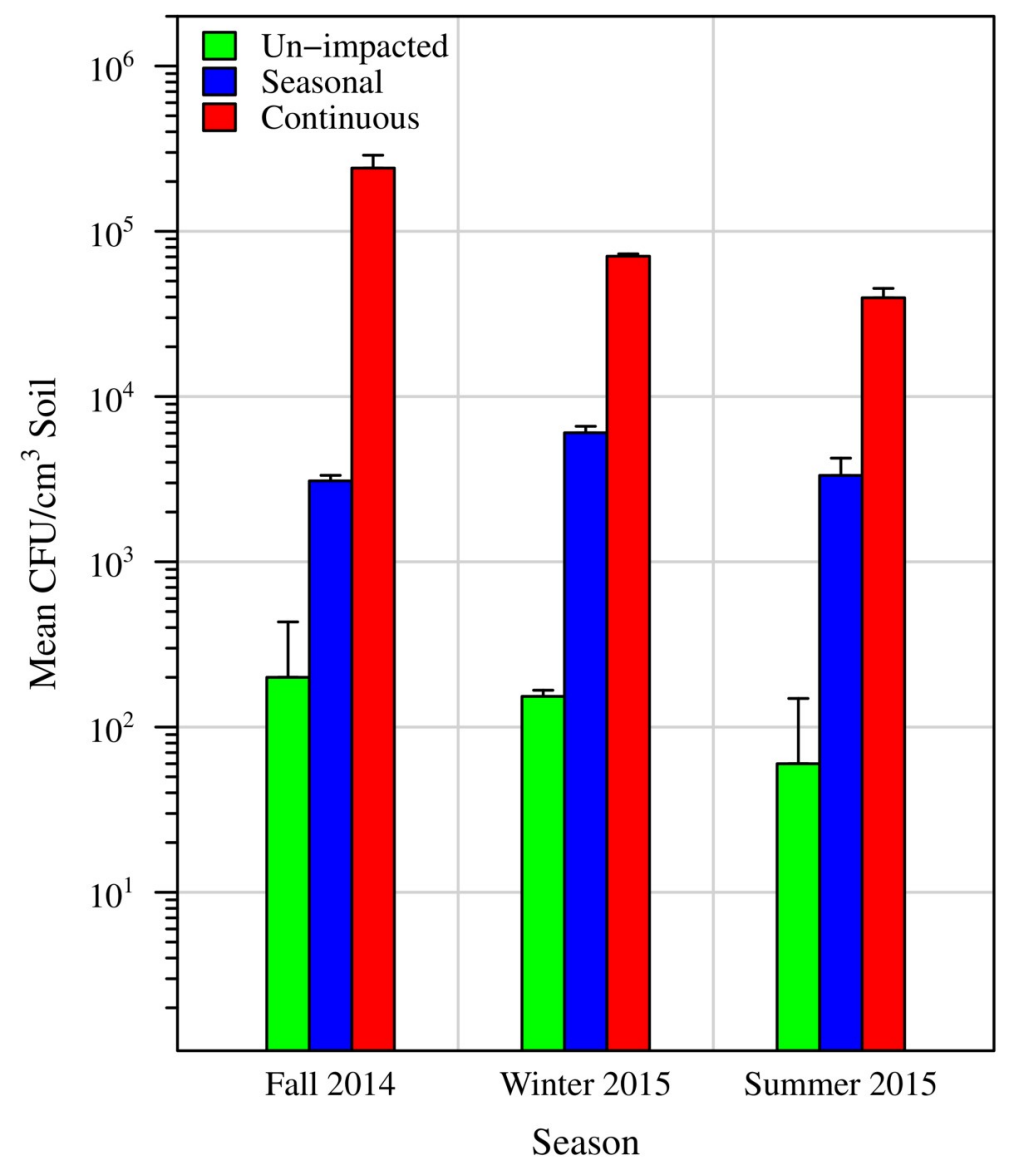
Number of colony forming units of halophiles at the different sites. Soils collected in November 2014, February 2015, and June 2015 were resuspended in HB broth supplemented with 2 M NaCl, serial dilutions plated onto HB agar plates supplemented with 2 M NaCl, and plates incubated for 48 hours at 37°C. Colonies were counted and colony forming units normalized to 1 cm^3^ of soil. The seasonal salt impacted site (blue) harbors significantly higher numbers of halophiles of at least one order of magnitude compared to the un-impacted site (green) at all seasons. Continuous salt exposure enhances the differences as the site that is continuously impacted (red) has at least one order of magnitude more halophiles than the seasonally impacted site (green). The y-axis of the plot is a logarithmic scale. Standard deviations are indicated by error bars.

We quantified halophiles over time in soil samples collected from the un-impacted, seasonally impacted, and continuously impacted sites. Soil was suspended in HB broth supplemented with 2 M NaCl, diluted as appropriate, and plated in triplicate onto 2 M NaCl HB media plates. After a 48 hour incubation period, colonies were counted. The continuously impacted site had the highest colony counts, ranging from 39,600 ± 5,546 (standard deviation [SD]) CFU/cm^3^ soil in summer to 241,333 ± 47,258 (SD) CFU/cm^3^ soil in fall, followed by the seasonally impacted site with counts ranging from 3,087 ± 247 (SD) CFU/cm^3^ soil in fall to 6,033 ± 571 (SD) CFU/cm^3^ soil in winter. Colony counts at the un-impacted site were several orders of magnitude lower, ranging from 60 ± 87 (SD) CFU/cm^3^ soil in summer to 200 ± 231 (SD) CFU/cm^3^ soil in fall (Fig 4). In each season, colony counts were significantly higher at the road salt impacted sites, compared to the un-impacted site (adjusted P-values of the seasonal and un-impacted site for fall and summer = 0.0104, and winter = 0.0069; adjusted P-values of the continuously and un-impacted site for fall and summer = 0.0005, and winter = 0.0003; 2-sided Conover-Iman test with Holm-Bonferroni correction). Similarly, the continuously impacted site had significantly higher colony counts than the seasonally impacted site (adjusted P-values for fall and summer = 0.0052, and winter = 0.0035) (Fig 4).

### Amplicon sequencing of the 16S rRNA gene

We assessed the microbial community compositions of salt and soil samples collected in winter 2013 and soil samples collected in the fall 2014, winter 2015, and summer 2015. A total of 458,725 OTUs were classified at the domain level. The majority of the sequences were assigned to the Bacteria (99.66%), with 0.34% of the sequences assigned to the Archaea. Among the Bacteria, 52 phyla were identified. The most abundant phylum was the *Proteobacteria* (36.59%), followed by the *Bacteroidetes* and *Verrucomicrobia* (17.86% and 10.84% of all reads, respectively). Among the Archaea, 4 phyla were present: *Euryarchaeota* (0.322%), *Nanoarchaeaeota* (0.016%), *Thaumarchaeota* (0.004%), and *Crenarchaeota* (0.001%) (S1 Table).

**Fig 5:**
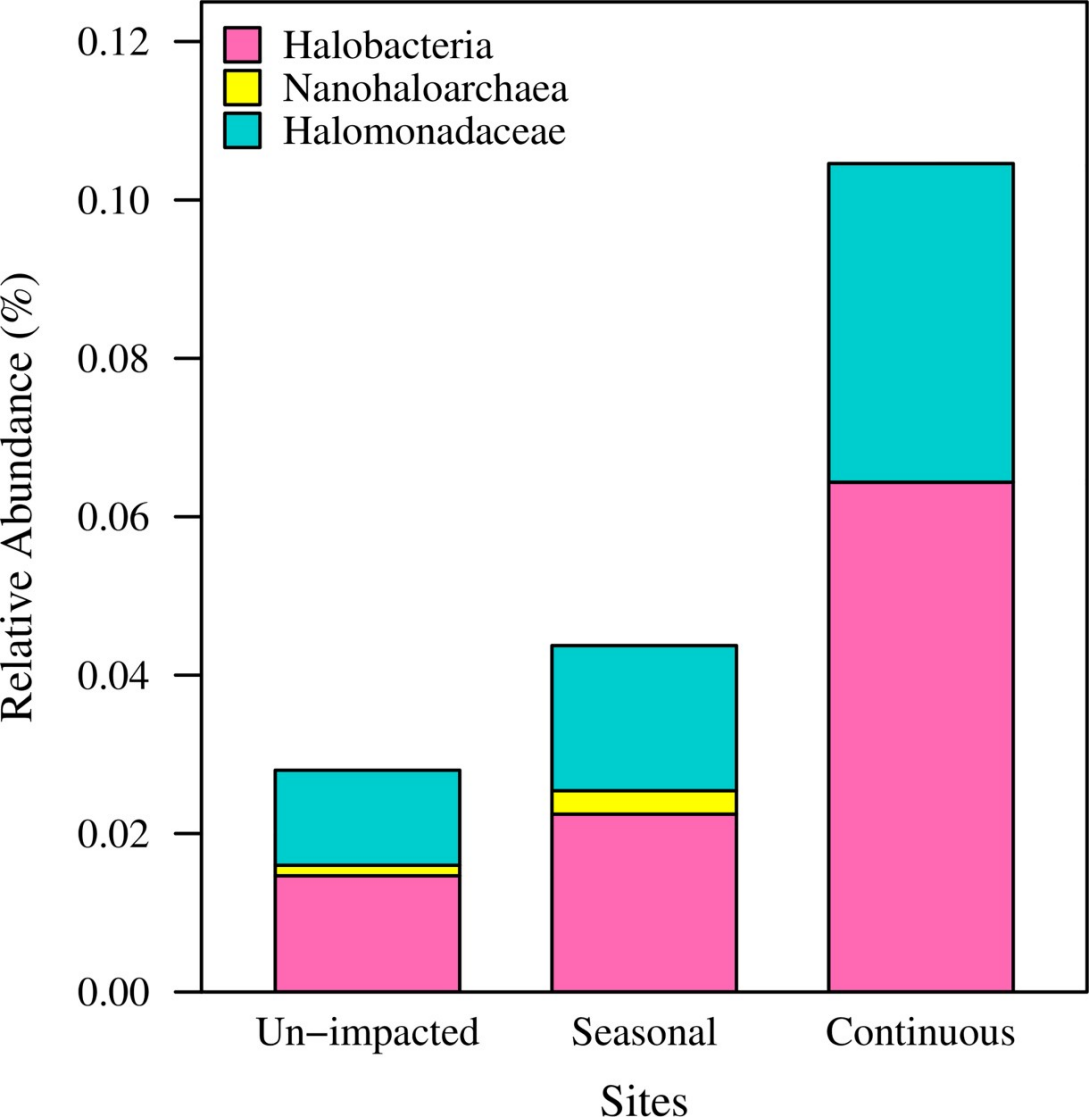
Relative abundance of representative halophiles in soil samples collected in 2014 and 2015. Amplicon sequencing of the 16S rRNA gene identified 3 prokaryotic groups comprised of predominately halophilic members that were present at the sites. The abundance of these groups was significantly higher at the continuously impacted site (adjusted P-values = 8.21 x 10^−10^ and 2.96 x 10^−9^, respectively; 2-sided Fisher’s exact test with Holm-Bonferroni correction). Note that *Nanohaloarchaea* were not found at the continuously impacted site.

**Fig 6:**
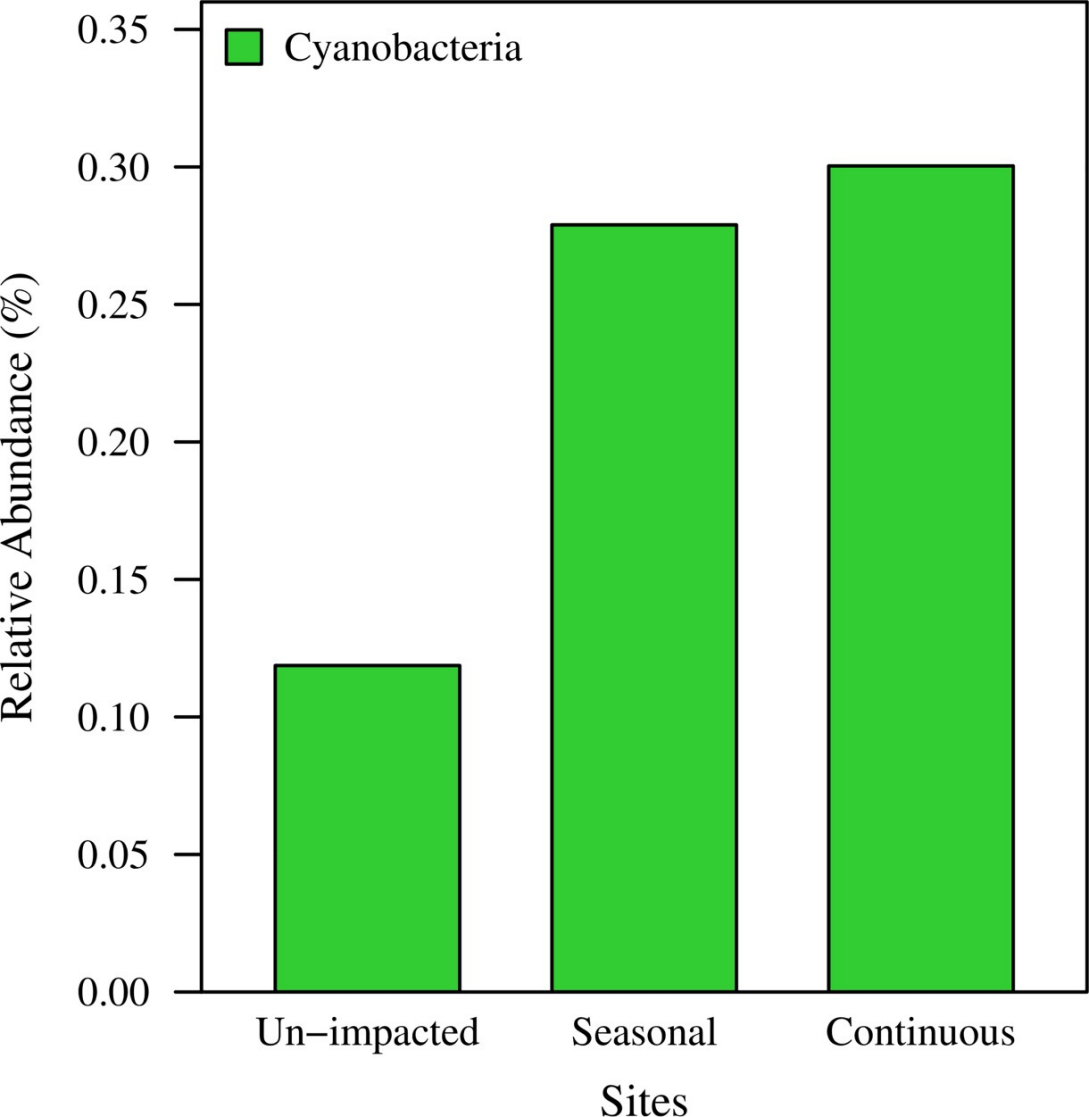
Relative abundance of *Cyanobacteria* in soil samples collected in 2014 and 2015. Based on amplicon sequencing of the 16S rRNA gene *Cyanobacteria* abundance was significantly higher at the seasonally and continuously impacted sites (adjusted P-values = 2.32 x 10^−15^ and 1.45 x 10^−16^, respectively; 2-sided Fisher’s exact test with Holm-Bonferroni correction).

If, as we hypothesized, halophiles become persistent members of the soil community, more halophilic organisms should be identified in road salt impacted soils. We therefore compared the abundance of representative halophiles identified in soil collected in fall 2014, as well as winter and summer 2015 at the 3 sites. These include Archaea belonging to the classes *Haloarchaeaceae*/*Halobacteria* and *Nanohaloarchaea*, as well as Bacteria of the order *Halanaerobiales*, and the family *Halomonadaceae* [37, 65–69]. Members of the *Halanaerobiale*s were not detected in the 2014/15 soil samples. *Haloarchaea/Halobacteria* and *Halomonadaceae* were found at all sites. *Nanohaloarchaea* were found at the seasonally impacted and at the un-impacted sites. The continuously impacted site had significantly more halophiles than the un-impacted and seasonally impacted sites (adjusted P-values = 8.21 x 10^−10^ and 2.96 x 10^−9^, respectively; 2-sided Fisher’s exact test with Holm-Bonferroni correction) (Fig 5). Similarly, we observed that *Cyanobacteria* were significantly more prevalent at the seasonal and continuously impacted sites (adjusted P-values = 2.32 x 10^−15^ and 1.45 x 10^−16^, respectively; 2-sided Fisher’s exact test with Holm-Bonferroni correction) (Fig 6).

## Discussion

Our data show that seasonal exposure to road salt leads to an increased presence of halophilic microorganisms, even months after road salt exposure. This finding is supported by our semi-quantitative spot test analysis (Fig 3), as well as by a quantitative measure of colony forming units retrieved from each site (Fig 4). These observations are corroborated by amplicon sequencing of the 16S rRNA gene depicted in Fig 5 and S1 Table. Altogether, this evidence shows the significant impact of road salt exposure on soil microbial communities, leading to a persistent and increased presence of halophilic and/or halotolerant microorganisms.

The use of road salt for de-icing has increased by orders of magnitude over the past decades [2, 9] (Fig 1). This trend is predicted to continue due to climate change effects [12]. Recent models have shown that the decline of Arctic sea ice is leading to changes in wind stream patterns resulting in more persistent snow and ice storms [70–72]. This includes more frequent polar vortex states leading to cold extremes during mid to late winters in mid-latitudes [73]. In addition, population growth and continuing urbanization are leading to an expanding network of roads and highways. Both factors will contribute to increased use of de-icing agents for the foreseeable future and consequently result in microbial communities with more halotolerant and/or halophilic species most likely beyond what we observed in this study.

Road salt pollution and the associated shift in microbial communities have wide-reaching implications for municipalities as they strive to mitigate nutrient loading and effect pollutant reductions in urban runoff. Among the practices used to reduce nutrient input in streams and lakes from urban (and agricultural) runoff are the installation of retention basins, rain gardens, and the expansion of riparian buffers. All these best management practices will, however, potentially be impacted by road salt runoff and buildup. In the laboratory setting, Endreny et al. [21] observed these impacts in their study of microbial communities in bioretention media. After exposure to artificial storm water supplemented with an equivalent of 26.4 mM NaCl (935 mg/L Cl), they reported a reduction in the ability of microbial communities to retain NO_3_^-^ or PO_4_. Mesocosm studies also showed that the ecology of storm water retention ponds is constrained by environmentally-relevant concentrations of road salt [74].

Similar to what was observed in mesocosm studies, it is conceivable that the observed changes in microbial communities in soils may result in a reduction or alteration of their ecosystem functions. Therefore, the reported reduced water retention of impacted soils [22], in concert with the possible reduced capacity of nutrient removal of salt contaminated soils and retention media, may impede the ability of storm water management structures to reduce nutrient input into urban water bodies. Increased salinity in combination with high nutrient levels in aquatic environments has been shown to lead to more frequent occurrence of algae blooms, especially cyanobacterial blooms, with increased release of cyanotoxins and considerable environmental and public health concerns [24–33]. In addition to the indirect effects that microbial communities of salt contaminated soils may have, the increased sodium levels in fresh water reservoirs may pose a direct threat to human health as sodium levels continue to rise in affected urban fresh water reservoirs [7, 17, 18, 75, 76]. In this context, our results raise concerns regarding the possibility of toxic cyanobacteria resulting from increased salinity in soils (Fig 6).

Our results demonstrate that halophilic microorganisms become persistent members of microbial communities as a result of salting roads for de-icing during winter months. In order to quantify increases in salinity and halophiles in the environment, we have developed and evaluated a facile and rapid spot test that utilizes a customized media formulation for capturing and cultivating halophilic microorganisms. Moreover, we show using this assay, that soils impacted by road salt harbor 10-fold more halophiles than un-impacted soils. As such, the spot test has the potential to be a valuable tool to survey and evaluate salt pollution of soils.

## Supporting information

S1 Table Abundance of prokaryotes at the domain and phylum level. (XLSX)
